# Correction: B Cell: T Cell Interactions Occur within Hepatic Granulomas during Experimental Visceral Leishmaniasis

**DOI:** 10.1371/journal.pone.0199184

**Published:** 2018-06-12

**Authors:** The PLOS ONE Staff

## Notice of Republication

This article was republished on May 29, 2018 to correct a technical error present in the original article acceptance date. The publisher apologizes for the error. Please download this article again to view the corrected version. The originally published, uncorrected article and the republished, corrected article are provided here for reference.

## Supporting information

S1 File Originally published, uncorrected article. (PDF)

S2 File Republished, corrected article. (PDF)
