# Supplementary material for: Correction: B Cell: T Cell Interactions Occur within Hepatic Granulomas during Experimental Visceral Leishmaniasis
Source: PLoS One. 2018 Jun 12;13(6):e0199184. doi: 10.1371/journal.pone.0199184 (PMC5997302; doi:10.1371/journal.pone.0199184)
Supplement: S1 File — (PDF) [file pone.0199184.s001.pdf]

OPEN ACCESS Freely available online

PLoS one

B Cell: T Cell Interactions Occur within Hepatic Granulomas during Experimental Visceral Leishmaniasis

John W. J. Moore, Lynette Beattie, Jane E. Dalton, Benjamin M. J. Owens¤a, Asher Maroof¤b, Mark C. Coles, Paul M. Kaye*

Centre for Immunology and Infection, Hull York Medical School and Department of Biology, University of York, Heslington, York, United Kingdom

Abstract

Hepatic resistance to *Leishmania donovani* infection in mice is associated with the development of granulomas, in which a variety of lymphoid and non-lymphoid populations accumulate. Although previous studies have identified B cells in hepatic granulomas and functional studies in B cell-deficient mice have suggested a role for B cells in the control of experimental visceral leishmaniasis, little is known about the behaviour of B cells in the granuloma microenvironment. Here, we first compared the hepatic B cell population in infected mice, where ≈60% of B cells are located within granulomas, with that of naïve mice. In infected mice, there was a small increase in $mIgM^{lo}mIgD^{+}$ mature B2 cells, but no enrichment of B cells with regulatory phenotype or function compared to the naïve hepatic B cell population, as assessed by CD1d and CD5 expression and by IL-10 production. Using 2-photon microscopy to quantify the entire intra-granuloma B cell population, in conjunction with the adoptive transfer of polyclonal and HEL-specific BCR-transgenic B cells isolated from *L. donovani*-infected mice, we demonstrated that B cells accumulate in granulomas over time in an antigen-independent manner. Intra-vital dynamic imaging was used to demonstrate that within the polyclonal B cell population obtained from *L. donovani*-infected mice, the frequency of B cells that made multiple long contacts with endogenous T cells was greater than that observed using HEL-specific B cells obtained from the same inflammatory environment. These data indicate, therefore, that a subset of this polyclonal B cell population is capable of making cognate interactions with T cells within this unique environment, and provide the first insights into the dynamics of B cells within an inflammatory site.

Citation: Moore JWJ, Beattie L, Dalton JE, Owens BMJ, Maroof A, et al. (2012) B Cell: T Cell Interactions Occur within Hepatic Granulomas during Experimental Visceral Leishmaniasis. PLoS ONE 7(3): e34143. doi:10.1371/journal.pone.0034143

Editor: Ben L. Kelly, Louisiana State University, United States of America

Received December 7, 2011; **Accepted** February 30, 2012; **Published** March 30, 2012

Copyright: © 2012 Moore et al. This is an open-access article distributed under the terms of the Creative Commons Attribution License, which permits unrestricted use, distribution, and reproduction in any medium, provided the original author and source are credited.

Funding: This work was supported by grants to PMK from the UK Medical Research Council and the Wellcome Trust. The funders had no role in study design, data collection and analysis, decision to publish, or preparation of the manuscript.

Competing Interests: The authors have declared that no competing interests exist.

* E-mail: paul.kaye@york.ac.uk

¤a Current address: Translational Gastroenterology Unit, Experimental Medicine Division, Nuffield Department of Medicine, University of Oxford, Oxford, United Kingdom
¤b Current address: GlaxoSmithkline Biologicals, Hamilton, Montana, United States of America

Introduction

B cells have many diverse roles in immunity extending far beyond their ability to produce antibodies. B cell-derived LTα1β2 plays a role in i) the differentiation of follicular dendritic cells (FDCs), mesenchymal cells that subsequently produce B cell survival factors and follicle-homing chemokines to maintain follicular architecture and function in lymphoid tissues [1] and ii) in infection-induced remodelling of this compartment [2]. During the early stages of an immune response, B cells function as APC and interact in a cognate manner with $CD4^{+}$ T cells to facilitate their own activation and differentiation into antibody secreting cells [3], and within the follicle, the interaction between B cells and follicular helper T cells is essential for survival and to rescue and expand high affinity class-switched B cells [4,5,6]. During this cross-talk, B cells also contribute cytokines or other factors that facilitate $CD4^{+}$ T cell functional differentiation [7]. In addition, a growing number of studies also implicate B cells, directly or indirectly, as negative regulators of T cell immunity through IL-10-dependent and IL-10-independent mechanisms [8,9,10,11,12,13]. A number of recent studies have exploited intra-vital 2-photon microscopy to dissect the dynamic behaviour of B cells in the follicular and extra-follicular environments of peripheral lymph nodes, defining many of the dynamic features associated with cognate B cell-T cell and B cell-DC interactions [14,15,16,17,18,19]. However, in spite of the common occurrence of B cells at inflammatory sites induced by infection and autoimmune disease [15,20,21], there is little known about the dynamic behaviour of B cells outside of peripheral lymphoid tissues.

The role of B cells in regulating immunity to leishmaniasis has been most extensively studied in models of cutaneous leishmaniasis, but with conflicting findings. Treatment with anti-IgM showed that susceptibility to *L. major* in BALB/c mice is B-cell dependent, possibly mediated by regulatory T cell induction by B cells [22,23], whereas in resistant C3H/HeN mice, B cells contribute to resistance [24]. Ronet et al. used B cell transfer experiments to show that the impact of B cells on susceptibility to *L. major* infection was parasite strain specific [25] and studies in T cell-reconstituted *scid* mice indicated that co-transfer of B cells could promote disease in a non-cognate manner [26]. Other studies however showed that B cell-deficient μMT mice did not exhibit any defects in T cell priming and polarisation [27]. In

addition, in another study, IL-7 treatment lead to B cell expansion, accelerated lesion growth and reduced survival [28]. Most recently, IL-10-producing B cells have been found in BALB/c mice infected with *L. major* and proposed to shape Th2 development [12]. In a different model of cutaneous leishmaniasis, infection of B cell-deficient JhD mice with *L. amazonensis* led to delayed lesion development, and these mice displayed decreased $CD4^+$ T cell recruitment to skin granulomas and decreased $CD4^+$ T cell responses, suggesting that B cells may play a role in priming the immune response against *L. amazonensis* infection [29].

In experimental visceral leishmaniasis (EVL), early studies identified infrequent $B220^+$ B cells in the hepatic granulomas of BALB/c mice infected with *L. donovani*, as well as B cells lying adjacent to infected Kupffer cells in infected athymic (nu/nu) BALB/c mice [30]. An inhibitory role for B cells in host resistance was indicated by studies of *L. donovani* infected B cell-deficient B6.μMT mice, which showed that these mice had accelerated granuloma formation and reduced hepatic parasite burden compared to B6 mice. Resistance could not be reversed by serum-transfer [31]. Furthermore, B6.μMT mice also had exaggerated hepatic neutrophil recruitment and suffered severe tissue pathology, suggesting that B cells may also play a role in protecting against host-mediated damage. More recently, polyclonally activated B cells appearing early after *L. infantum* infection and producing IgM have been implicated in disease exacerbation [9]. Although the induction of myeloid cell-derived IL-10 by immune complexes has been well-described [32], this study indicated a distinct IL-10-independent role for C5 activation and C5aR (CD88) signalling in IgM-mediated immune regulation [9].

In addition to providing information on the cellular mechanism of host immunity to *L. donovani*, the hepatic granulomas formed during EVL provide an excellent model system to study the dynamics of cellular interactions outside of peripheral lymphoid tissues, combining a variety of attributes including small size, complex cellular composition and accessibility to intra-vital imaging [33,34,35]. Here, we address whether B cell recruitment to hepatic granulomas is a function of phenotype, time, antigen-specificity and/or the development of tertiary lymphoid organ (TLO) characteristics. We show that B cells accumulate over time p.i., but enter and exit granulomas with relative ease independently of antigen specificity and without enrichment of regulatory B cells. By comparing the dynamics of polyclonal B cells and transgenic B cells with an irrelevant specificity, we demonstrate significant differences in their interactions with intra-granulomas T cells and the existence of cognate B:T cell interactions within the granuloma microenvironment. These data provide the first glimpse of the behaviour of B cells in an inflammatory setting and should inform the design of future imaging studies in this and other disease settings.

Results

B cells traffic into *L. donovani*-induced hepatic granulomas

In BALB/c mice, intra-granuloma $B220^+$ cells have been described [30]. Similarly, in B6 mice, $B220^+$ B cells were mostly observed in the periphery of granulomas (**Figure 1A and B**), with number being directly proportional to granuloma cross-sectional area ($r = 0.4756$; $p<0.05$; **Figure 1C**). Although by flow cytometry, ~90% of $B220^+$ cells co-expressed CD19, $B220^+$ $CD19^-$ cells expressing CD11c and/or γδ TCR were also present (**Figure 1D**). To more selectively examine B cells, we infected $CD19^{cre}xR26^{eYFP}xVa^{DsRed}$.A mice (herein called B^{green}/T^{red} mice; **Figure 2**), where eYFP is expressed on all cells that have expressed CD19 or are derived from $CD19^+$ cells [36], and DsRed is expressed on T cells under the hCD2 locus control region [37] (**Figure S1**). We also employed 2-photon microscopy to capture the entire 3-dimensional cellular composition of individual granulomas (**Figure 2**; [33]), to avoid the sampling limitations of conventional thin sections. $CD19^+$ cells were readily detected at d14 p.i. (**Figure 2A**), though outnumbered 12:1 by T cells (**Figure 2D**). At d21 and day 28 p.i., B cells were more abundant ($62.9 \pm 1.6\%$ of all hepatic B cells being found within granulomas; $n = 95$ FOV, $n = 3$ mice at d21) and the T:B cell ratio within granulomas was decreased (**Figure 2B–D**). In some but not all granulomas, B cells aggregated into small clusters (**Figure 2E and F**). Although B cell aggregation is often associated with the development of TLOs and associated FDCs [38], we could not detect ectopic $CD21/CD35^+$ FDCs within hepatic granulomas (**Figure 2G**).

Phenotype of hepatic B cells in infected mice

As hepatic granulomas cannot be isolated in a reproducible way, total hepatic B cell populations (of which ~60% represent intra-granuloma B cells, see above) were studied by flow cytometry. In naïve livers, $CD19^+$ $mIgM^{hi}mIgD^{lo/-}$ (transitional/MZ), $mIgM^{hi}mIgD^+$ (T2) and $mIgM^{lo}mIgD^+$ (mature) B cells were all found, in addition to a small proportion of $mIgM^{lo/-}mIgD^{lo/-}$ cells, which likely represent class-switched mature B cells. In infected mice, the frequency of T2 B cells was reduced with a commensurate increase in $mIgM^{lo}mIgD^+$ mature B cells (**Figure 3A and B**). Indicative of activation, we found a 1.7 ± 0.2 fold-increase in MFI for MHCII expression on intra-hepatic B cells from infected compared to naïve mice. In contrast, CD40 expression was unchanged (**Figure 3C and D**). Plasma cells ($CD138^+$) were not specifically recruited into the hepatic microenvironment of infected mice and were diluted out in the hepatic B cell pool of infected mice (**Figure 3E and F**).

Expression of CD1d and CD5 in naïve and infected mice was minimal (<3%), again with evidence of dilution by mature B cells in infected mice (**Figure 4A–C**). After PMA, ionomycin and LPS stimulation [39] negligible levels of IFN-γ and IL-4 were detected in $CD19^+$ cells (**Figure 4D, E and H**). $CD19^+$ cells produced TNF, but the frequency was lower in infected compared to naïve mice (**Figure 4F, H and I**). A small fraction of $CD19^+$ cells also made IL-10 and accumulated detectable levels of IL-10 mRNA, but hepatic B cells from infected mice did not have an elevated capacity for IL-10 production (**Figure 4G, H and I**). Collectively, these data suggest that most intra-hepatic B cells in infected mice have a phenotype consistent with recruited T2 and mature re-circulating B2 cells that have been non-specifically activated by the cytokine environment in infected mice.

Adoptively transferred B cells are recruited to hepatic granulomas

To confirm that peripheral B cells could be recruited into granulomas from the circulation, we co-transferred $CD19^+$ B cells (>90% purity; data not shown) from infected and naïve mice that expressed similar levels of the liver homing receptor CCR6 [40], into d21-infected VaDsRed (herein called T^{red}) mice (**Figure 5A–D**). 12 h following B cell transfer, labelled B cells originating from both naïve and infected mice were found in similar numbers (**Figure 5E–G**). Hence, within 12 h of transfer, B cells from naïve as well as infected mice are capable of being recruited from the periphery into pre-existing granulomas.

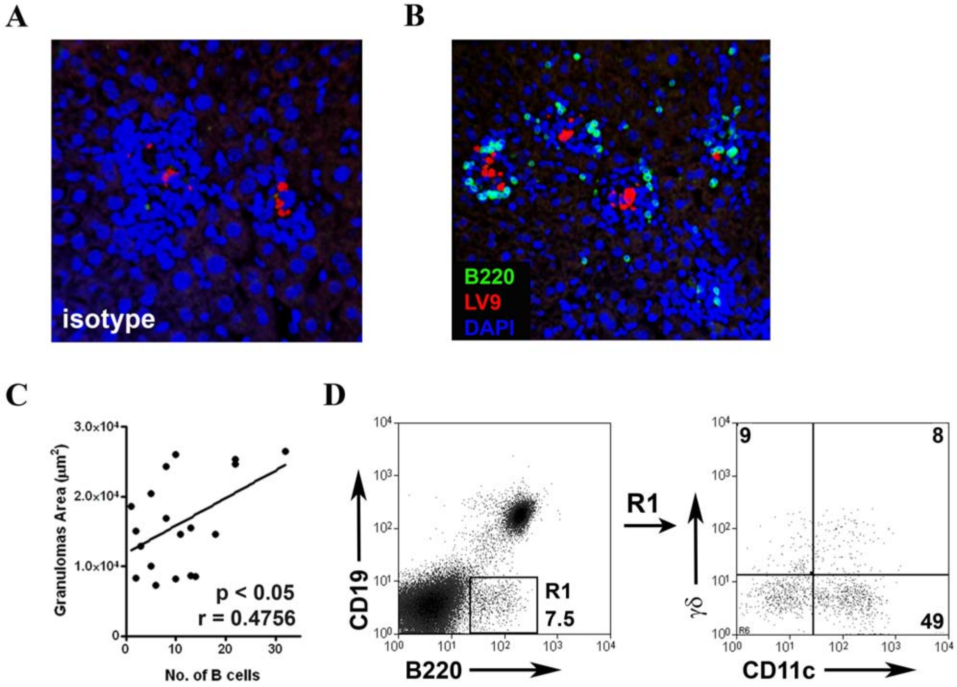

Figure 1. B cells are present in *L. donovani*-induced hepatic granulomas. A and B. C57BL/6 mice (n = 3) were infected with 3×10^7 TdTomato transgenic *L. donovani* amastigotes (red) and on day 21 p.i., liver sections were prepared and stained using an isotype control mAb or with B220 (green) mAb. Sections were counterstained with DAPI (blue). **C.** Correlation between granuloma cross-sectional area and number of intra-granuloma $B220^+$ cells. Each symbol represents a single granuloma. **D.** Hepatic mononuclear cells were labeled with CD19 and B220, and $B220^+CD19^-$ cells (R1) were subsequently analyzed for CD11c and γδ TCR.
doi:10.1371/journal.pone.0034143.g001

Dynamic imaging of B cells in hepatic granulomas

As the behaviour of B cells outside of the LN is unknown, we first examined the dynamics of an endogenous B cell population in granulomas of infected B^{green}/T^{red} mice. B cells moved freely, with average velocity of 4.2±0.3 µm/min (**Figure 6A**), similar to granuloma T cells [33,35] and B cells in lymphoid tissue [14,17,18,19]. Under static imaging conditions, T:B conjugates were readily observed, sometimes being associated with collagen fibres that traversed the granuloma (**Figure 6B and C**).

To examine these interactions further, we developed a co-transfer model allowing simultaneous analysis of two independent B cell populations within the same granulomas, thus controlling for heterogeneity in granuloma size, maturation status and/or cellular composition [33]. We used MD-4 transgenic B cells with defined but un-related antigen specificity (hen egg lysozyme; HEL) as a comparator for immune B cells (i.e. polyclonal B cells from infected mice). To ensure that all B cells has been exposed to the same inflammatory environment prior to transfer, we infected MD-4 (CD45.2)→B6.CD45.1 bone marrow micro-chimeras (**Figure 7A**) and at d21 p.i., $CD19^+$ B cells were sort purified (>98% purity) on the basis of CD45.1/2 expression (**Figure 7B**). MD-4 B and immune B cells were labelled and co-transferred into d21-infected T^{red} recipient mice. 12 h post transfer, intra-vital imaging was performed (**Figure 7C and D**). Similar numbers of immune B cells and MD-4 B cells were detected within granulomas at d21 p.i. (**Figure 7E**), further confirming that antigen-recognition per se was not required for B cells to be recruited into *L. donovani*-induced hepatic granulomas.

For 134 immune B cells and 121 MD-4 B cells, we determined individual displacement, track length and average velocity and calculated the displacement rate and meandering index (**Figure 8A–E**). By none of these criteria could we distinguish the behaviour of immune B cells from MD-4 B cells. This result was expected given the highly motile nature of B cell-T cell conjugates seen in lymphoid tissue [17] and the similar degree of mobility of granuloma B cells (**Figure 6A**) and T cells [33,35]. We then examined the ability of B cells to engage with endogenous T cells, using the study of Qi et al as a template for our analysis and to allow ready comparison with data obtained using MD-4 B cells in LN [17]. We analysed 1089 individual contacts between MD-4 B cells and endogenous T cells. The average contact duration of 2.81±0.11 min was similar to that observed for non-cognate interactions in the LN follicle. Nevertheless, at a population level, MD-4 B cells made a substantial number of contacts that were longer than expected for non-cognate interactions, mostly between 3 and 10 min of duration but often extending for 30 min (**Figure 9A**). In contrast to MD-4 B cells, the average contact time for immune B cells was 4.02±0.19 min (n = 756 contacts, $P<0.0001$; **Figure 9A**), indicating that at a population level, these immune B cells had sustained engagement with T cells compared to MD-4 B cells. Stratifying the data according to contact time per incidence confirmed that there was a significantly greater trend for polyclonal B cells to make contacts of longer duration than those made by MD-4 B cells (**Figure 9B**; $\chi^2 = 34.9$, df = 4, $p<0.0001$). We next analysed each individual B cell for the duration of contacts that it made with individual T cells. A range of behaviour was observed, from repeated short interactions to single long contacts that spanned the entire imaging window. Single frame images taken from the imaging videos, and contact plots (where each period of contact is indicated by a white box) are shown for two MD-4 B cells displaying different behaviour during a 30 min imaging window (**Figure 9C and Video S1**). For each B cell shown, there were also periods when simultaneous contact was made with multiple T cells (shown as adjacent white boxes in

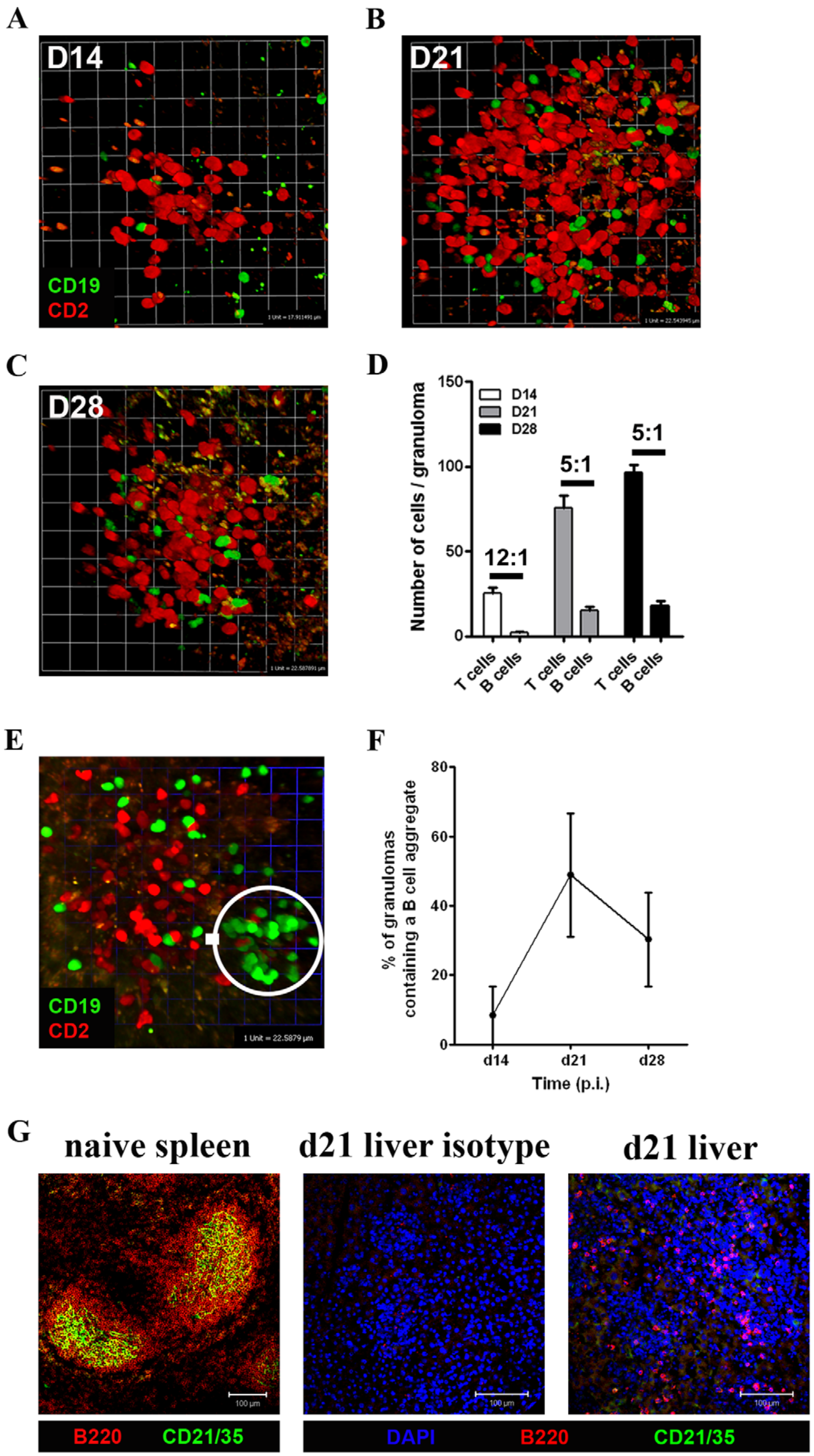
A
D14
CD19
CD2
B
D21
C
D28
D
150
100
50
0
Number of cells / granuloma
D14
D21
D28
12:1
5:1
5:1
T cells
B cells
T cells
B cells
T cells
B cells
E
CD19
CD2
1 Unit = 22.5879 µm
F
80
60
40
20
0
% of granulomas containing a B cell aggregate
d14
d21
d28
Time (p.i.)
G
naive spleen
d21 liver isotype
d21 liver
100 µm
B220
CD21/35
DAPI
B220
CD21/35

Figure 2. The recruitment of B cells to *L. donovani*-induced hepatic granulomas. B^{green}/T^{red} mice (n = 6) were infected with *L. donovani* and at d14 (**A**), d21 (**B**) and d28 (**C**) p.i. liver explants were imaged using 2-photon microscopy to visualize T cells and B cells. **D.** Number of T cells and B cells at d14 (open bars) d21 (grey bars) and d28 (black bars) p.i. derived from 25–35 hepatic granulomas per time point from 2 mice per group. Data are shown as mean ± SEM along with the T cell: B cell ratio. **E.** B cells aggregate in hepatic granuloma. **F.** The number of B cell aggregates per granuloma was determined from 25–35 granulomas (mean ± SEM). **G.** C57BL/6 mice were infected with *L. donovani* (n = 3). Frozen sections were labeled with B220 (red), CD21/35 (green) and counterstained with DAPI (blue, except left panel). 60 hepatic granulomas were imaged. Control staining of spleen was performed (left panel).
doi:10.1371/journal.pone.0034143.g002

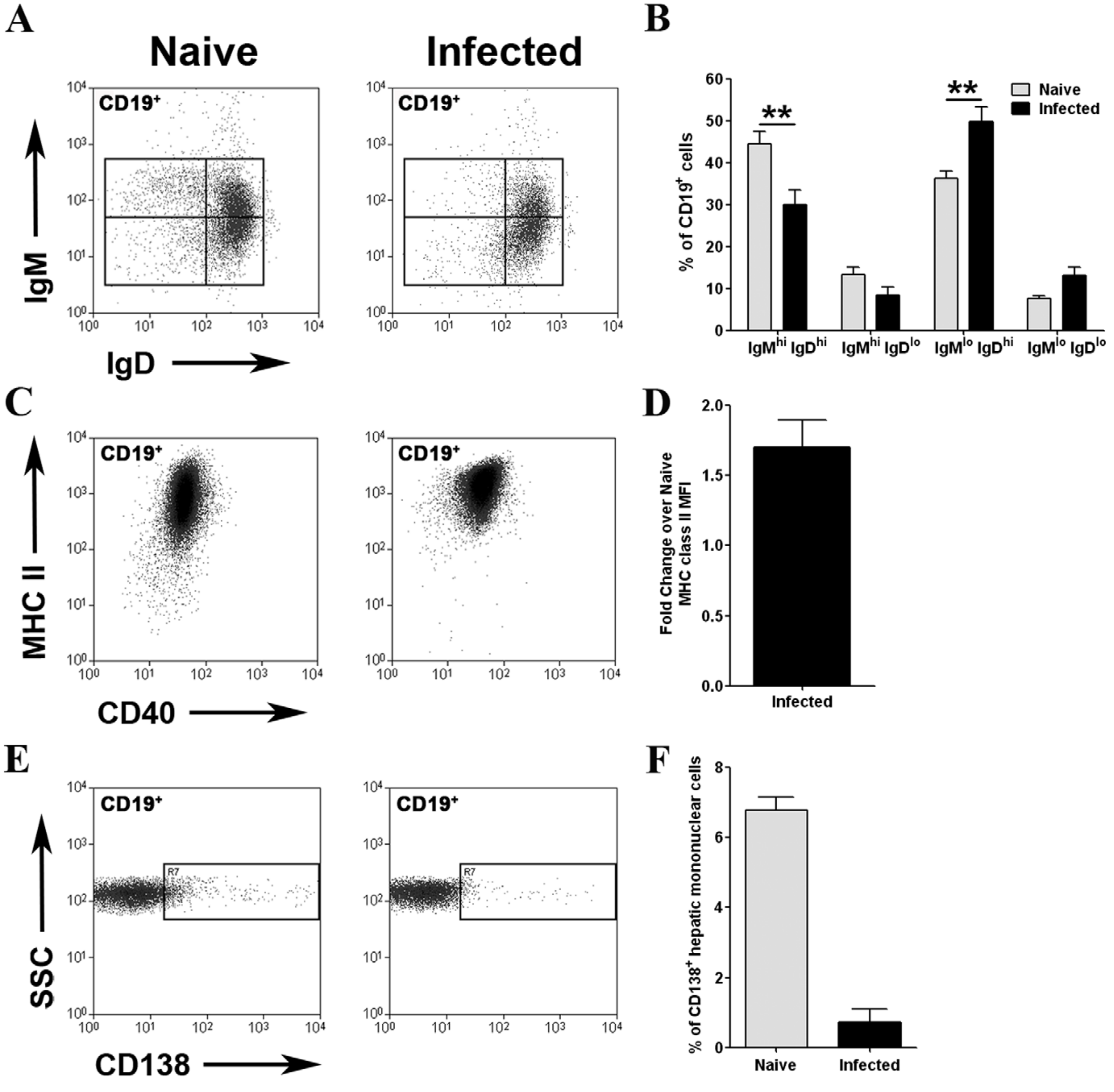

Figure 3. Phenotype of hepatic B cells in *L. donovani*-infected mice. A. Hepatic mononuclear cells from naïve and d21 *L. donovani*-infected C57BL/6 mice were stained for CD19 along with membrane IgM and membrane IgD. **B.** Frequency of T1, T2, mature and class-switched B cells in naïve (grey bars) and infected (black bars) mice. Data represents the mean ± SEM for 5 independent experiments with cells pooled from 3 mice per experiment (**, $p<0.01$). **C.** $CD19^+$ cells were stained for MHCII and CD40. **D.** Data are shown as fold change in MFI compared to naïve (mean ± SEM from 5 independent experiments). **E.** Expression of CD138 on $CD19^+$ cells from naïve and infected mice. **F.** Data are shown as % $CD19^+$ cells in naïve (grey bars) and infected (black bars) mice expressing CD138.
doi:10.1371/journal.pone.0034143.g003

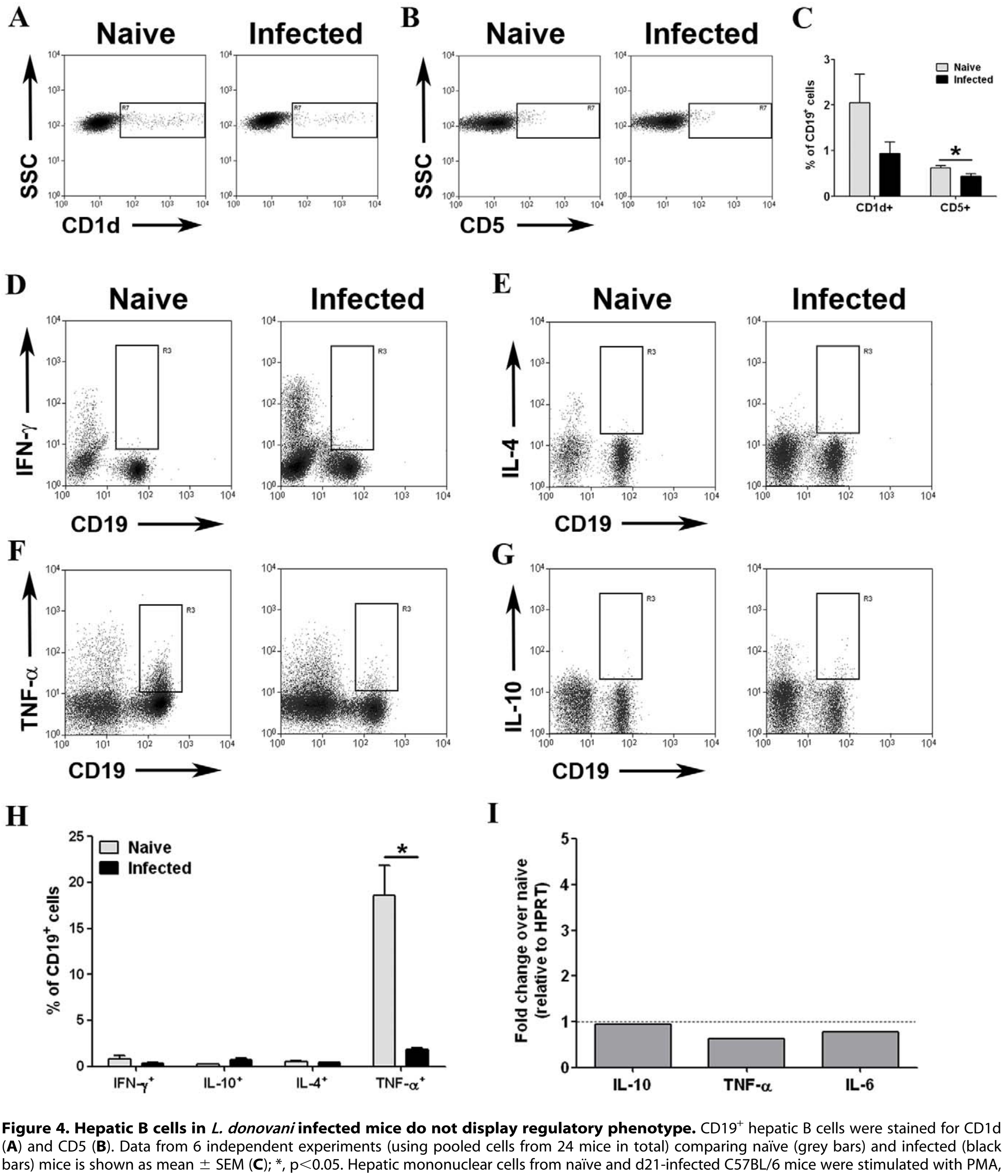

Figure 4. Hepatic B cells in *L. donovani* infected mice do not display regulatory phenotype. $CD19^+$ hepatic B cells were stained for CD1d (**A**) and CD5 (**B**). Data from 6 independent experiments (using pooled cells from 24 mice in total) comparing naïve (grey bars) and infected (black bars) mice is shown as mean ± SEM (**C**); *, $p<0.05$. Hepatic mononuclear cells from naïve and d21-infected C57BL/6 mice were stimulated with PMA, ionomycin and LPS and stained for IFNγ (**D**), IL-4 (**E**), TNF (**F**) and IL-10 (**G**). Data are from 3 independent experiments, with cells pooled from 3–4 mice per experiment. **H.** Data from 3 independent experiments comparing naïve (grey bars) with infected (black bars) mice is shown as mean ± SEM % of $CD19^+$ cells positive for each cytokine. *, $p<0.05$. **I.** Hepatic $CD19^+$ B cells from naïve and d21-infected C57BL/6 mice were FACS purified and mRNA accumulation for IL-10, TNF and IL-6 was determined by quantitative RT-PCR. Data are shown as fold change compared to mRNA accumulation in naïve mice and represent mean ± SEM for 3 mice assayed in duplicate.

doi:10.1371/journal.pone.0034143.g004

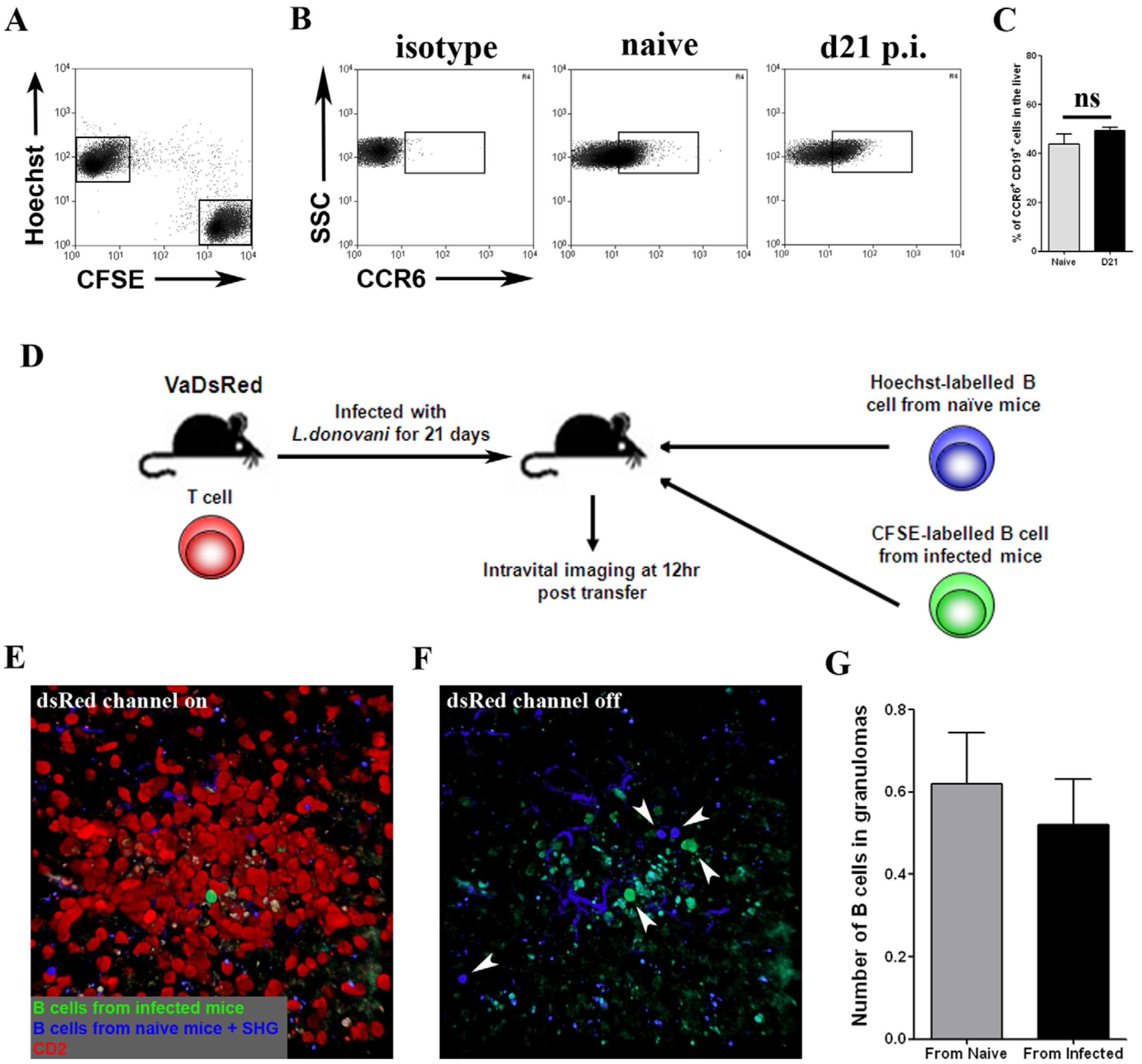

Figure 5. Naïve and immune B cells are recruited into hepatic granulomas. A. B cells were CD19$^+$ MACS-purified (>90% B220$^+$) from naïve and d21-infected mice and labeled with Hoechst and CFSE, respectively. **B.** CCR6 expression on CD19$^+$ B cells from naïve and d21-infected mice (n = 4). **C.** Data showing frequency of CCR6$^+$CD19$^+$ B cells in naïve (grey bars) and infected (black bars) mice. **D.** Protocol for mixed adoptive transfer of labeled B cells into T^{red} mice. **E and F.** Maximum projection image of transferred labeled B cells in granuloma of T^{red} mouse, shown with (E) and without (F) DsRed channel switched on. **G.** Number of labeled naïve (grey bars) and immune (black bars) B cells found per granuloma. Data are shown as mean ± SEM for 50 granulomas derived from imaging livers of two mice.
doi:10.1371/journal.pone.0034143.g005

Figure 9C). However, we did not observe any B cell division within granulomas during this study. When summated to provide an accumulated total contact time for each B cell, the total contact time for any individual B cell also varied widely (from less than 1 min to over 80 min). It was difficult, however, to discern any significant difference between the behaviour of the immune and MD-4 B cell populations by this approach (**Figure 9D**). Nevertheless, when we analysed the percentage of short (<3 min) vs. long (3–40 min) contacts made by each B cell population, immune B cells had a significant bias towards making long term contacts compared to the MD-4 B cells ($p<0.01$; **Figure 9E**). Although demonstrating a clear difference in behaviour at the population level, due to the variability in behaviour of the MD-4 B cells it was not possible to determine with any degree of statistical certainty the absolute frequency of immune B cells that engaged in these cognate interactions with granuloma T cells.

Discussion

B cells are a notable feature of inflammation, yet little is known about their behaviour and ability to interact with T cells outside of the lymphoid follicle. Using a model of hepatic granulomatous

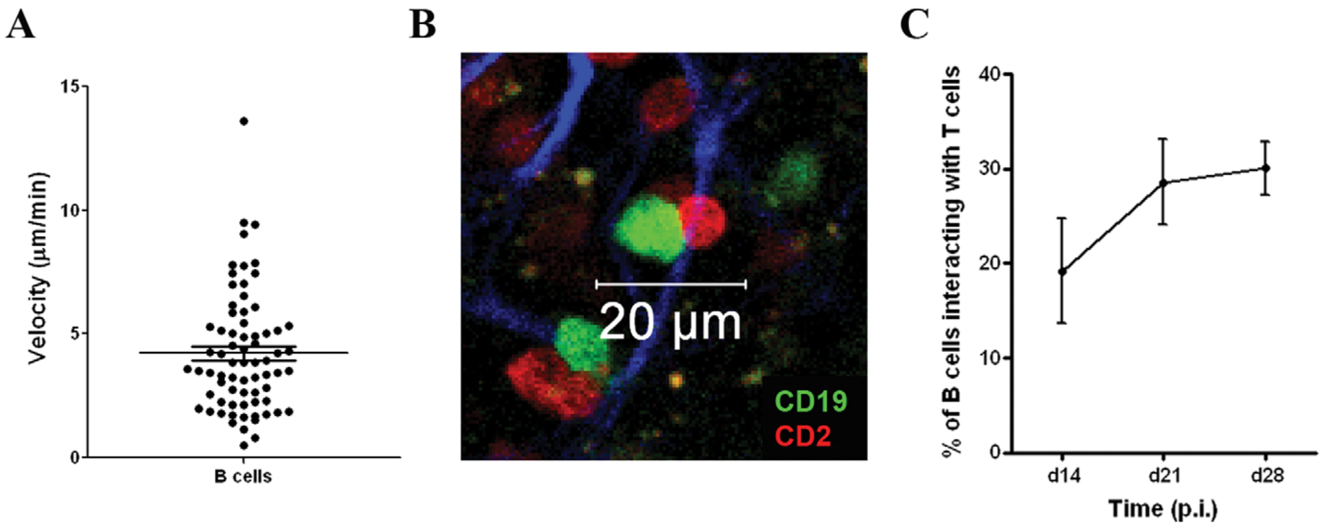

Figure 6. Endogenous B cell behavior in hepatic granulomas. A. B cell velocity in granulomas of d21-infected B^{green}/T^{red} mice. Each symbol represents an individual B cell. Bar shows median velocity calculated from 71 B cells in 12 granulomas imaged in two mice. Liver explants tissue from B^{green}/T^{red} mice was imaged using 2-photon microscopy and B cell-T cell contacts (**B**) identified by static imaging. The second harmonic signal is also visible (blue). Data represents the mean ± SEM % B cells interacting with T cells (**C**) and were derived from 25 and 35 granulomas from 2 mice per time point.
doi:10.1371/journal.pone.0034143.g006

inflammation, our data provide the first direct evidence that peripheral B cells are recruited into inflammatory sites independently of their antigen specificity, exhibit highly motile behaviour and have the capacity to interact with intra-granuloma T cells. Although activation of B cells by inflammation or other BCR-independent signals increased the duration of contact with T cells compared to that described for naïve follicular B cells, we also found evidence that a small proportion of intra-granuloma B cells interact in a manner consistent with cognate interactions with T cells.

The granulomas we have studied in B6 mice do not appear to have taken on characteristics of TLOs. Although B cells occasionally occur in small aggregates, no FDC networks were detected and there was limited 'organisation' beyond the clustering of B cells. Most B cells, as well as T cells, were highly motile and were often seen to traverse the entire granuloma, further suggesting the absence of the compartmentalisation traditionally associated with TLOs [38]. In addition, we have previously noted that DC, implicated in TLO generation [41], are also sparse within these granulomas [33]. Hence, the *L. donovani*-induced granuloma represents an inflammatory infiltrate with a structure loosely determined by the nidus of infected Kupffer cell(s), and even compared to granulomas induced by BCG [35], they appear to be more loosely organised. To what extent granuloma organisation and local cellular dynamics may vary in other strains of mice with less effective host immunity and hence prolonged parasite burden, and/or in the hepatic granulomas associated with human VL, remains to be determined.

Ex vivo analysis of granuloma B cells was complicated by difficulties in reproducibly isolating granulomas from the surrounding non-involved hepatic tissue, in a way that was not selective from granulomas at particular stages of differentiation. However, as approximately 60% of hepatic B cells are found within granulomas, we believe that any major changes in phenotype between the hepatic B cells in naïve mice and those of infected mice would have been discernable, even if we could not attribute them directly to B cells within granulomas. Whereas we could readily demonstrate that hepatic B cells from infected mice had enhanced MHCII expression and a more mature phenotype based on relative sIgM and sIgD staining, our analysis did not detect any evidence to show enrichment for B cells with regulatory phenotype in the liver of infected mice. Studies *in L. infantum*-infected BALB/c mice have previously identified a population of IL-10 producing $CD19^+$ B cells in the LN and spleen [9] and $B220^+$ cells in the liver [42] of infected mice. This discrepancy may reflect differences in methodology (e.g. antigen restimulation [9] vs. polyclonal activation, measurement of secreted cytokine in supernatants [42] vs. intracellular flow cytometry, use of B220 vs. CD19, or the kinetics of the response studied). Alternatively, this may reflect a mouse strain difference, as we have also previously noted that NK cell-derived IL-10 and the frequency of $IFN\gamma^+IL\text{-}10^+$ $CD4^+$ T cells ([43] and Maroof, unpublished) is greater in BALB/c mice than B6 mice infected for similar periods of time. That it was not possible to accurately identify antigen-specific B cells by phenotype or cytokine production is not surprising given that the frequency of B cells responsive to soluble *Leishmania* antigen in *L. infantum*-infected mice is only ~1–2% [9]. We saw a small trend towards an increase in the frequency of class-switched B cells in the liver of infected mice, but this did not reach statistical significance. Together with our data showing that B cells from naïve and infected mice have similar levels of CCR6 expression and home similarly into granulomas within 12 h of adoptive transfer, the simplest interpretation is that most granuloma B cells represent B cells in the circulation that encounter and enter granulomas where these structures occlude sinusoidal flow. Although we have observed B cells leaving granulomas, and presumably re-entering the circulation, the relatively short imaging time used in this study was not optimal for determining how long each individual B cell remained within a specific granuloma.

Previous studies of B cell-T cell interactions in peripheral lymphoid tissue have characterised B:DC and B:$CD4^+$ T cell interactions after emigration from HEV and within the follicular environment, respectively [17,18]. Using co-transferred cognate and non-cognate combinations of TCR transgenic T cells and BCR transgenic B cells, these authors demonstrated that contacts

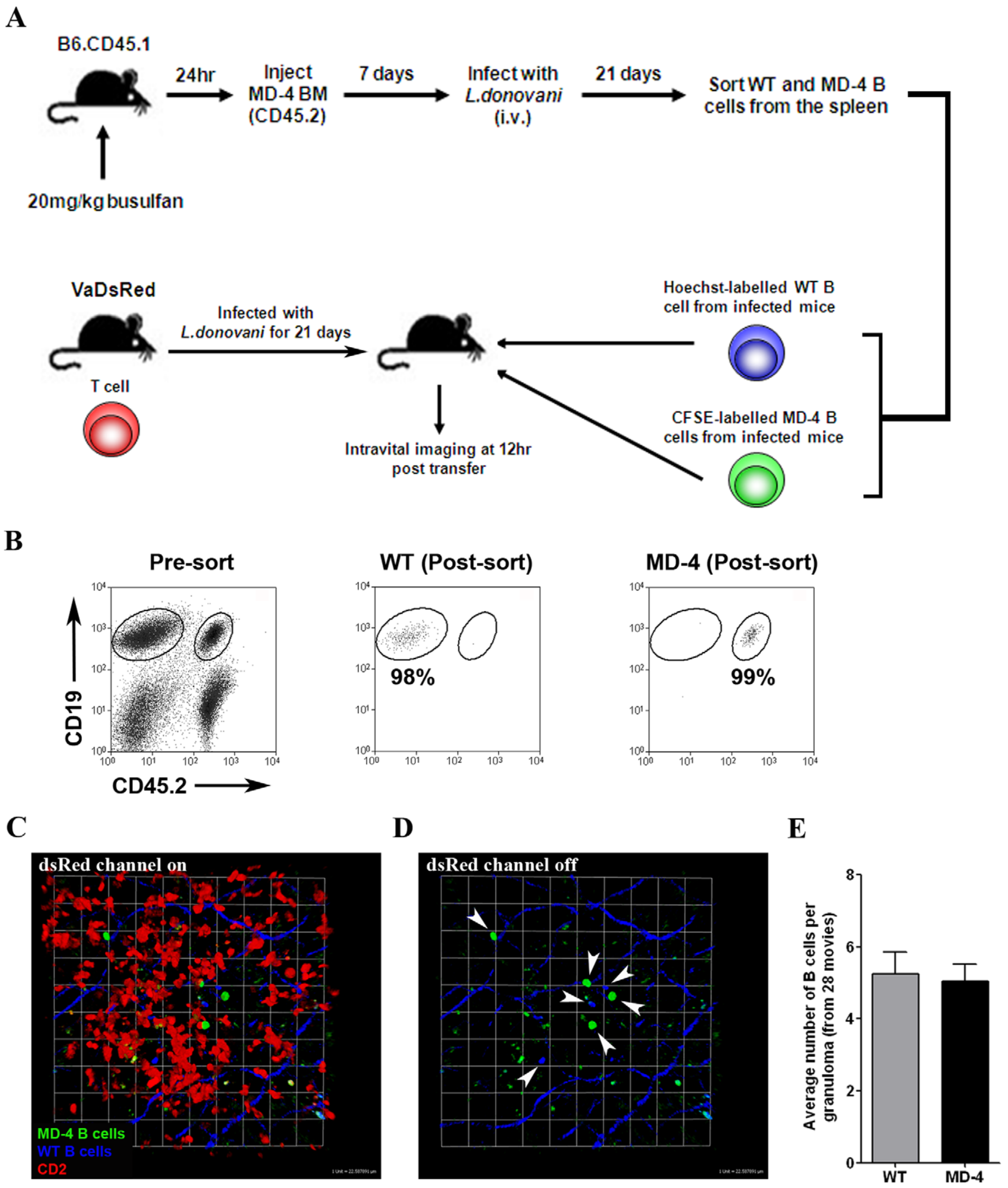

Figure 7. Role of antigen specificity in B cell recruitment to granulomas. A. Protocol for mixed adoptive transfer. **B.** Pre and post sort purity of adoptively transferred B cells. **C and D.** Maximum projection image of transferred labeled MD-4 (green) and immune (blue) B cells in granuloma of T^{red} mouse 12 h post transfer, shown with (C) and without (D) DsRed channel switched on. **E.** Number of recruited MD-4 (black bars) and immune (grey bar) B cells found in granulomas 12 h post transfer. Data represent mean ± SEM B cell per granuloma derived from 28 granulomas from two mice.

doi:10.1371/journal.pone.0034143.g007

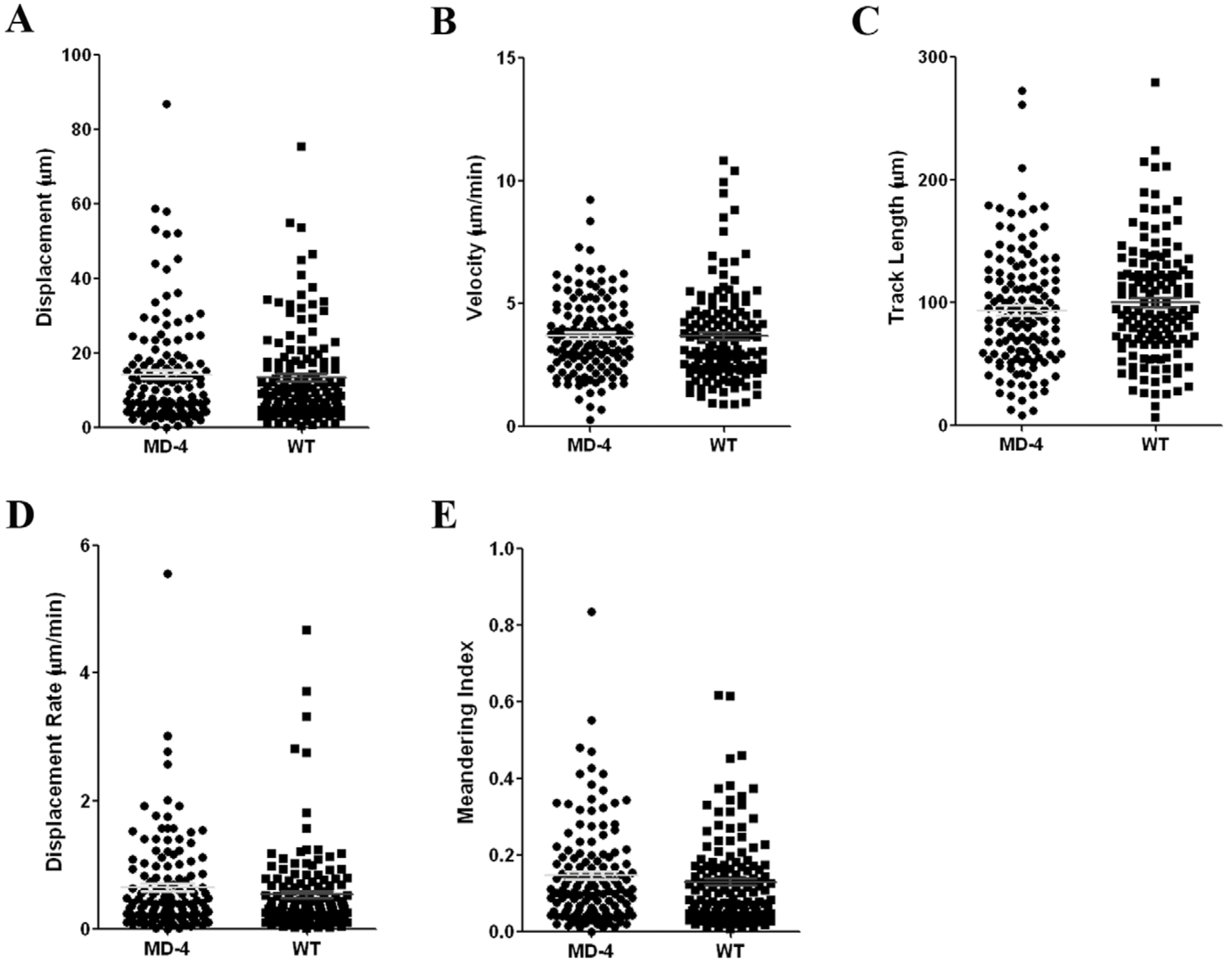

Figure 8. Dynamic behavior of MD-4 and immune B cells in the granuloma microenvironment. Labeled MD-4 and immune B cells were transferred into d21-infected T^{red} mice as described in Figure 7. At 12–30 h post adoptive transfer, time lapse z-stack videos were prepared, and each B cell was manually tracked in Volocity software to determine (**A**) displacement, (**B**) velocity, (**C**) track length, (**D**) displacement rate and (**E**) meandering index. Data are shown for each individual B cell analyzed within 28 granulomas from two mice.
doi:10.1371/journal.pone.0034143.g008

with CD4$^+$ T cells lasting longer than 3 min were indicative of antigen-specific interactions. We observed a similarly short average contact time per incidence when following the behaviour of HEL-specific MD-4 cells in granulomas, in spite of a limitation of our model being that that the identification of endogenous T cells was based on DsRed expressed from the hCD2 LCR, which includes both CD4$^+$ and CD8$^+$ T cells (and some NK cells) [44]. B cells also make relatively short non-cognate interactions with DC in LN, whereas cognate interactions between these two cells last an average of 10 min and may extend to >45 min [18]. Although DC are relatively sparse in granulomas and do not appear to contain parasites at an appreciable frequency or abundance [33], we have not directly examined whether B cells interact with DC in this environment. Our data do indicate, however, that many of the characteristics of B cell dynamics observed in LN appear to hold true when examining these cells in an inflammatory and non-lymphoid setting.

McMahon-Pratt and colleagues [9] have recently re-examined the well-known phenomenon of polyclonal B cell activation in EVL, with important bearings on the interpretation of our data. First, as a comparator B cell population for our dynamic imaging studies on immune B cells, we used HEL-specific MD-4 B cells that had been parked for 21 d in infected B6 mice. Given that the frequency of OVA, chromatin and TNP-specific antibody-secreting B cells increased 5–8-fold over 14 d in infected BALB/c mice [9], it might be expected that MD-4 cells might also be subject to similar polyclonal activation signals. This could provide an explanation for why we found that although at a population level, the contact time for MD-4 cells with endogenous T cells was similar to that expected for naïve MD-4 cells [17], our data showed a much greater degree of spread of contact time per incidence (up to 30 min) than previously observed, where no non-cognate contacts lasted more than 5 min. Alternatively, and with potentially similar consequences, it has also been proposed that bystander B cells may acquire BCR from antigen-activated B cells through membrane transfer [45]. The greater spread of contact times observed between granuloma T cells and MD-4 B cells in our studies made it difficult to obtain and apply a definitive threshold for cognate and non-cognate interactions when analyzing the immune B cell population. In addition we could

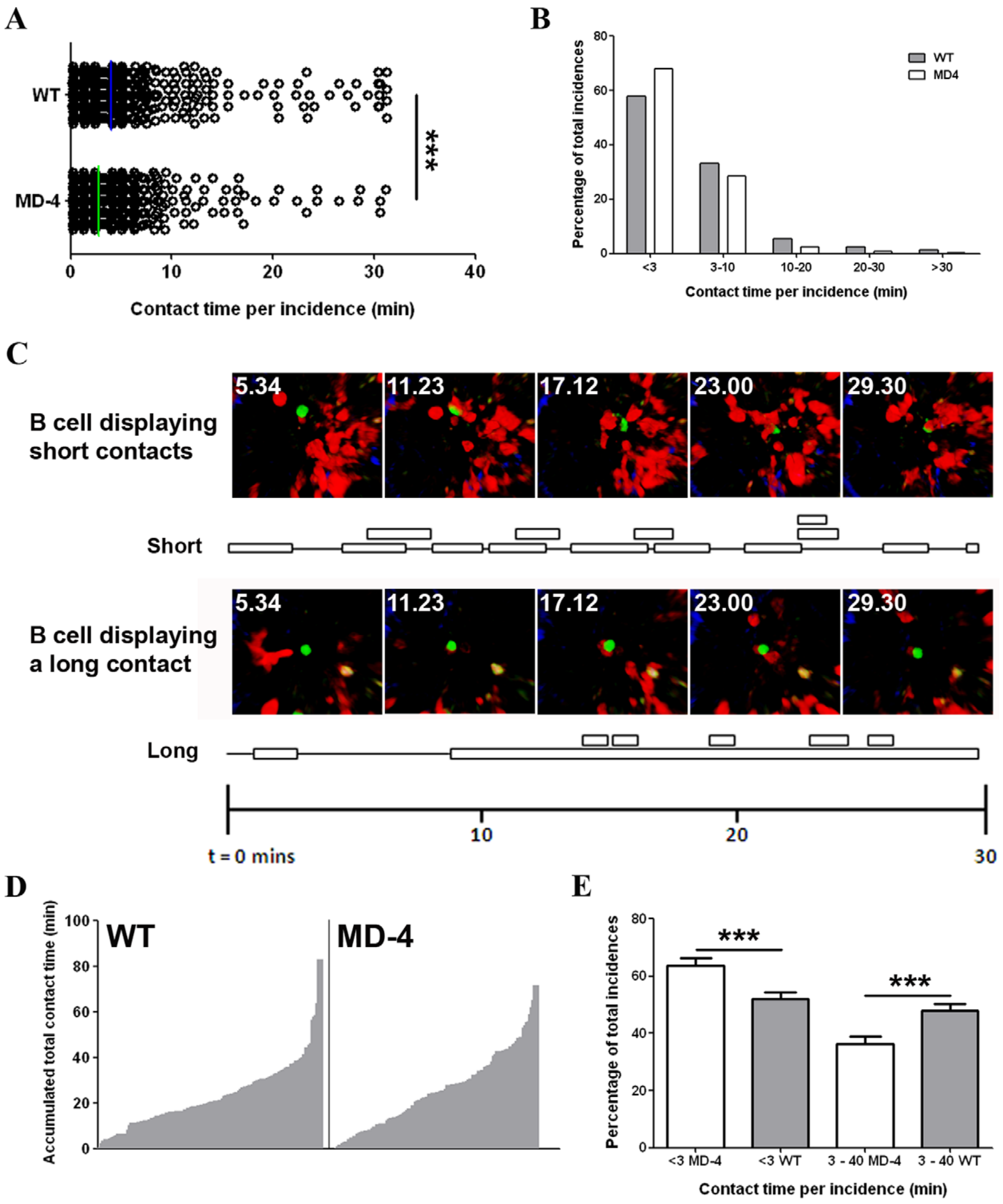

Figure 9. B cells in granulomas make contacts with endogenous T cells. A. MD-4 and immune B cells were evaluated for contacts with DsRed+ cells in 28 granulomas imaged from two *L. donovani*-infected T^{red} mice. The duration of all individual contacts is shown as a separate symbol. **B.** The frequency of contacts of different duration is shown for MD-4 (open bars) and immune (grey bars) B cells; $P<0.001$). **C.** Single frames from Video S1 showing two immune B cells making short (top images) or long (bottom images) contacts with DsRed+ cells. Contact plot is shown below, where each box represents a contact period and adjacent boxes indicate where multiple DsRed+ cells are bound simultaneously. **D.** The total time each individual B cell (grey bars) spends interacting with any granuloma T cell during the imaging period (i.e. the accumulated total contact time) is shown. Note that due to interactions with multiple T cells, some B cells show an accumulated total contact time longer than the imaging time of 30 min. **E.** Distribution of contact times for each individual MD-4 (open bars) or immune (grey bars) B cell. Data are expressed as frequency of total incidences for each B cell that involved short (<3 min) or long (>3 min) contact with an endogenous DsRed+ cell. **, $p<0.01$.
doi:10.1371/journal.pone.0034143.g009

not retrospectively confirm whether B cells engaging in longer contacts displayed a different phenotype or activation status. It is likely that this technical limitation will also apply to any study of B cells in the environment of the *Leishmania* infected mouse, irrespective of additional manipulations that might be employed to further define the transferred populations of B cells. The extent of polyclonal activation is thus an important consideration for future imaging-based studies in this or other models of infection where polyclonal activation may occur.

In conclusion, we have shown that mature B cells are non-selectively recruited into hepatic granulomas during *L. donovani* infection, with no enrichment of 'regulatory' B cells, and that some engage in long-lasting interactions with T cells. Our data therefore provide the first illustration of the dynamic nature of B cells at an inflammatory site, whilst also suggesting caution in the interpretation of histopathologic data on lymphocyte recruitment in the absence of measures of antigen-specificity.

Methods

Ethics statement

All experiments were approved by the University of York Animal Procedures and Ethics Committee and performed under UK Home Office license ('Immunity and Immunopathology of Leishmaniasis' Ref # PPL 60/3708).

Mice and Infections

6–12 week old female C57BL6 mice (Charles River, Margate, UK), congenic B6.CD45.1, $CD19^{cre}R26R^{eYFP}Va^{DsRed}$ mice (B^{green}/T^{red}: National Institute for Medical Research, UK) and VaDsRed.B (T^{red}; NIMR, UK) were used. Femurs from MD-4 mice [46] backcrossed onto B6.μMT background were a kind gift of Dr G. Kassiotis (NIMR, UK). *Leishmania donovani* (LV9) and a transgenic line expressing TdTomato [47] were maintained in $B6.RAG1^{-/-}$ mice. 2–3×10^7 amastigotes were injected intravenously (i.v.) to initiate infection [48].

Intra-vital imaging

Intra-vital imaging was performed as described [33]. Briefly, mice were anaesthetised with ketamine (100 mg/kg), xylazine (10 mg/kg) and acepromazine (1.7 mg/kg), the left liver lobe was exteriorised and the animal placed on a custom made imaging platform. Images were acquired on an inverted LSM 510 multiphoton microscope (Carl Zeiss Microimaging) using a 36°C blacked-out environmental chamber (Solent Scientific, UK). For 4D analysis, 20–40 μM Z stacks were acquired with a Z distance of 2–3 μM approximately every 15–30 sec. Data were rendered and analysed using Volocity software (Improvision) and cell tracking performed manually, or automatically with manual checking. Cell-cell interactions were measured manually by determining the presence of cell-cell contacts in a 3D rendering on a frame-per-frame basis for each movie. The times of initial contact and the time that contact ended was recorded and the difference was calculated manually.

Analysis of explant tissue

Freshly removed liver tissue was placed in 35 mm coverslip bottom Petri dishes (MatTek Corporation), and imaged with a 40×1.1 water immersion objective and fluorescence excitation provided by a Chameleon XR Ti:sapphire laser (Coherent) tuned to 872 nm. Granuloma volumes were calculated by drawing regions of interest in Volocity to obtain a 3D volume measurement in μm^3. Blocks mounted in OCT were stored at −80°C freezer until use. 10 μm cryosections of liver and spleen were stained using B220, CD35 and CD21 antibodies, and analysed using a Zeiss LSM 510.

Flow cytometry and cytokine analysis

Livers were perfused with ice-cold saline via the hepatic portal vein, and placed into ice-cold RPMI. Tissue was passed through a 100 μm strainer, washed twice (1400 rpm, 7 min), and hepatic mononuclear cells isolated on a 33% Percoll gradient. Hepatic mononuclear cells were resuspended in RPMI with 10% FCS, plated into 96-well plates (1–2×10^6 cells/well) and incubated in FACS buffer containing CD16/32 antibodies for 10 min on ice. After washing, cells were stained in 100 μl FACS buffer containing fluorochrome-labelled antibodies specific for IgM (R6-60.2), IgD (11-26c.2a) B220 (RA3-6B2), CD19 (1D3) CD11c (N418), TCR γδ (GL3) CD5 (53-7.3) CD1d (1B1), CD40 (1C10) or MHCII (M5/114.15.2) or appropriate isotype controls (all from eBioscience or BD Biosciences). For cytokine analysis, cells were plated into a 24-well plate (5×10^6 cells/well) with 50 ng/ml PMA, 500 ng/ml ionomycin and 10 μg/ml LPS at 37°C in 5% CO^2 for 3 hr. 10 μg/ml of Brefeldin A was added for a further 4 hr, before fixation in 2% PFA (10 min on ice), permeabilization (0.5% saponin) and staining IFNγ (XMG1.2), IL-4 (BVD6-24G2), IL-10 (JES5-16E3) and TNF (MP6-XT22). Labelled cells were analysed on a Cyan flow cytometer with Summit software (Beckman Coulter).

As required, B cells were isolated by positive selection using an LS MACS column according to manufacturer's instruction (Miltenyi), after incubation with biotin-conjugated anti-CD19 antibody and streptavidin-conjugated magnetic beads.

B cell adoptive transfer

MACS purified spleen cells or hepatic mononuclear cells were stained with FITC-conjugated anti-CD45.2 (as required) and PE-conjugated anti-CD19 and sorted on a MoFlo (Beckman Coulter). Purified B cells were resuspended (1–5×10^6 cells/ml) in either CFDA-SE (5 μM) or Hoechst 33342 (6 μM) for 8 min at 37°C followed by quenching with cold medium. After washing, labelled B cells were resuspended in RPMI (8×10^6–1×10^7 in 200 μl) and injected i.v., alone or in a 1:1 ratio.

Real time –RT-PCR

mRNA accumulation for cytokines was performed as described elsewhere [43]. Briefly, 1×10^5 sort-purified cells were re-suspended in RLT buffer and RNA isolated with RNeasy spin columns according to the manufacturer's instructions (Qiagen). Eluted RNA was converted to cDNA and cDNA stored at −20°C or used immediately. The RT-PCR mix consisted of SYBR Green, forward primer (1 μM) and reverse primer (1 μM) and reactions were preformed in MicroAmp Optical 96-well reaction plates using an ABI Prism 7000 RT-PCR system. RT-PCR data was analysed using the 7000 system SDS software

Statistical analysis

Parametric or non-parametric tests were selected according to the distribution of the raw data. Comparisons between experimental groups were performed using students t test or Mann-Whitney. Correlations were determined using the Pearson correlation test and distribution data analyzed by chi-squared test. All analyses were conducted using GraphPad InStat (version 3) software (GraphPad software, San Diego, California, US).

Supporting Information

Figure S1 Splenocytes from B^{green}/T^{red} mice were labeled with anti-CD3 and anti-B220 Ab. Dot plot shows gated $eYFP^{+}$ and $DsRed^{+}$ populations within the spleen and histograms their respective expression of B220 and CD3. Grey population in histogram represents $eYFP^{-}dsRed^{-}$ cells from left hand panel. Dotted line in histograms represents the B220/CD3 staining of the total population shown in left hand panel.
(TIF)

Video S1 Dynamic behavior of adoptively transferred CFSE-labelled MD-4 cells (green) in granulomas of *L. donovani*-infected T^{red} mice, showing example of short and long contact (indicated by arrows) with endogenous T cells. Second harmonic is shown in blue.
(MOV)

Acknowledgments

The authors thank Dr G. Kassiotis for MD-4 bone marrow, Jo Marrison and Peter O'Toole (Technology Facility, University of York) for microscopy advice and the staff of the Biological Services Facility for animal husbandry.

Author Contributions

Conceived and designed the experiments: JWJM LB MCC PMK. Performed the experiments: JWJM LB JED BMJO AM. Analyzed the data: JWJM LB JED BMJO AM PMK. Contributed reagents/materials/analysis tools: MCC. Wrote the paper: JWJM PMK. Supervised the study: PMK.

References

1. Ansel KM, Ngo VN, Hyman PL, Luther SA, Forster R, et al. (2000) A chemokine-driven positive feedback loop organizes lymphoid follicles. Nature 406: 309–314.
2. Kumar V, Scandella E, Danuser R, Onder L, Nitschke M, et al. (2010) Global lymphoid tissue remodeling during a viral infection is orchestrated by a B cell-lymphotoxin-dependent pathway. Blood 115: 4725–4733.
3. Rodriguez-Pinto D (2005) B cells as antigen presenting cells. Cell Immunol 238: 67–75.
4. Cannons JL, Qi H, Lu KT, Dutta M, Gomez-Rodriguez J, et al. (2010) Optimal germinal center responses require a multistage T cell:B cell adhesion process involving integrins, SLAM-associated protein, and CD84. Immunity 32: 253–265.
5. Nurieva RI, Chung Y (2010) Understanding the development and function of T follicular helper cells. Cell Mol Immunol 7: 190–197.
6. Pereira JP, Kelly LM, Cyster JG (2010) Finding the right niche: B-cell migration in the early phases of T-dependent antibody responses. Int Immunol 22: 413–419.
7. Crawford A, Macleod M, Schumacher T, Corlett L, Gray D (2006) Primary T cell expansion and differentiation in vivo requires antigen presentation by B cells. J Immunol 176: 3498–3506.
8. Buendia AJ, Del Rio L, Ortega N, Sanchez J, Gallego MC, et al. (2002) B-cell-deficient mice show an exacerbated inflammatory response in a model of Chlamydophila abortus infection. Infect Immun 70: 6911–6918.
9. Deak E, Jayakumar A, Cho KW, Goldsmith-Pestana K, Dondji B, et al. (2010) Murine visceral leishmaniasis: IgM and polyclonal B-cell activation lead to disease exacerbation. Eur J Immunol 40: 1355–1368.
10. Maglione PJ, Xu J, Chan J (2007) B cells moderate inflammatory progression and enhance bacterial containment upon pulmonary challenge with Mycobacterium tuberculosis. J Immunol 178: 7222–7234.
11. Moritoki Y, Zhang W, Tsuneyama K, Yoshida K, Wakabayashi K, et al. (2009) B cells suppress the inflammatory response in a mouse model of primary biliary cirrhosis. Gastroenterology 136: 1037–1047.
12. Ronet C, Hauyon-La Torre Y, Revaz-Breton M, Mastelic B, Tacchini-Cottier F, et al. (2010) Regulatory B cells shape the development of Th2 immune responses in BALB/c mice infected with Leishmania major through IL-10 production. J Immunol 184: 886–894.
13. Wilson MS, Taylor MD, O'Gorman MT, Balic A, Barr TA, et al. (2010) Helminth-induced CD19+CD23hi B cells modulate experimental allergic and autoimmune inflammation. Eur J Immunol 40: 1682–1696.
14. Gonzalez SF, Lukacs-Kornek V, Kuligowski MP, Pitcher LA, Degn SE, et al. (2010) Capture of influenza by medullary dendritic cells via SIGN-R1 is essential for humoral immunity in draining lymph nodes. Nat Immunol 11: 427–434.
15. Halle S, Dujardin HC, Bakocevic N, Fleige H, Danzer H, et al. (2009) Induced bronchus-associated lymphoid tissue serves as a general priming site for T cells and is maintained by dendritic cells. J Exp Med 206: 2593–2601.
16. Malissen B (2009) Revisiting the follicular helper T cell paradigm. Nat Immunol 10: 371–372.
17. Qi H, Cannons JL, Klauschen F, Schwartzberg PL, Germain RN (2008) SAP-controlled T-B cell interactions underlie germinal centre formation. Nature 455: 764–769.
18. Qi H, Egen JG, Huang AY, Germain RN (2006) Extrafollicular activation of lymph node B cells by antigen-bearing dendritic cells. Science 312: 1672–1676.
19. Suzuki K, Grigorova I, Phan TG, Kelly LM, Cyster JG (2009) Visualizing B cell capture of cognate antigen from follicular dendritic cells. J Exp Med 206: 1485–1493.
20. Greter M, Hofmann J, Becher B (2009) Neo-lymphoid aggregates in the adult liver can initiate potent cell-mediated immunity. PLoS Biol 7: e1000109.
21. Mueller A, Holl-Ulrich K, Lamprecht P, Gross WL (2008) Germinal centre-like structures in Wegener's granuloma: the morphological basis for autoimmunity? Rheumatology (Oxford) 47: 1111–1113.
22. Sacks DL (1988) B cell dependent T lymphocyte responses in leishmaniasis. Mem Inst Oswaldo Cruz 83 Suppl 1: 506–513.
23. Sacks DL, Scott PA, Asofsky R, Sher FA (1984) Cutaneous leishmaniasis in anti-IgM-treated mice: enhanced resistance due to functional depletion of a B cell-dependent T cell involved in the suppressor pathway. J Immunol 132: 2072–2077.
24. Scott P, Natovitz P, Sher A (1986) B lymphocytes are required for the generation of T cells that mediate healing of cutaneous leishmaniasis. J Immunol 137: 1017–1021.
25. Ronet C, Voigt H, Himmelrich H, Doucey MA, Hauyon-La Torre Y, et al. (2008) Leishmania major-specific B cells are necessary for Th2 cell development and susceptibility to L. major LV39 in BALB/c mice. J Immunol 180: 4825–4835.
26. Hoerauf A, Rollinghoff M, Solbach W (1996) Co-transfer of B cells converts resistance into susceptibility in T cell-reconstituted, Leishmania major-resistant C.B-17 scid mice by a non-cognate mechanism. Int Immunol 8: 1569–1575.
27. Brown DR, Reiner SL (1999) Polarized helper-T-cell responses against Leishmania major in the absence of B cells. Infect Immun 67: 266–270.
28. Gessner A, Will A, Vieth M, Schroppel K, Rollinghoff M (1995) Stimulation of B-cell lymphopoiesis by interleukin-7 leads to aggravation of murine leishmaniasis. Immunology 84: 416–422.
29. Wanasen N, Xin L, Soong L (2008) Pathogenic role of B cells and antibodies in murine Leishmania amazonensis infection. Int J Parasitol 38: 417–429.
30. McElrath MJ, Murray HW, Cohn ZA (1988) The dynamics of granuloma formation in experimental visceral leishmaniasis. J Exp Med 167: 1927–1937.
31. Smelt SC, Cotterell SE, Engwerda CR, Kaye PM (2000) B cell-deficient mice are highly resistant to Leishmania donovani infection, but develop neutrophil-mediated tissue pathology. J Immunol 164: 3681–3688.
32. Mosser DM, Edwards JP (2008) Exploring the full spectrum of macrophage activation. Nat Rev Immunol 8: 958–969.
33. Beattie L, Peltan A, Maroof A, Kirby A, Brown N, et al. (2010) Dynamic imaging of experimental Leishmania donovani-induced hepatic granulomas detects Kupffer cell-restricted antigen presentation to antigen-specific CD8 T cells. PLoS Pathog 6: e1000805.
34. Egen JG, Rothfuchs AG, Feng CG, Horwitz MA, Sher A, et al. (2011) Intravital imaging reveals limited antigen presentation and T cell effector function in mycobacterial granulomas. Immunity 34: 807–819.
35. Egen JG, Rothfuchs AG, Feng CG, Winter N, Sher A, et al. (2008) Macrophage and T cell dynamics during the development and disintegration of mycobacterial granulomas. Immunity 28: 271–284.
36. Rickert RC, Roes J, Rajewsky K (1997) B lymphocyte-specific, Cre-mediated mutagenesis in mice. Nucleic Acids Res 25: 1317–1318.
37. de Boer J, Williams A, Skavdis G, Harker N, Coles M, et al. (2003) Transgenic mice with hematopoietic and lymphoid specific expression of Cre. Eur J Immunol 33: 314–325.
38. Carragher DM, Rangel-Moreno J, Randall TD (2008) Ectopic lymphoid tissues and local immunity. Semin Immunol 20: 26–42.
39. Yanaba K, Bouaziz JD, Haas KM, Poe JC, Fujimoto M, et al. (2008) A regulatory B cell subset with a unique CD1dhiCD5+ phenotype controls T cell-dependent inflammatory responses. Immunity 28: 639–650.
40. Schutyser E, Struyf S, Van Damme J (2003) The CC chemokine CCL20 and its receptor CCR6. Cytokine Growth Factor Rev 14: 409–426.
41. GeurtsvanKessel CH, Willart MA, Bergen IM, van Rijt LS, Muskens F, et al. (2009) Dendritic cells are crucial for maintenance of tertiary lymphoid structures in the lung of influenza virus-infected mice. J Exp Med 206: 2339–2349.

42. Wilson ME, Sandor M, Blum AM, Young BM, Metwali A, et al. (1996) Local suppression of IFN-gamma in hepatic granulomas correlates with tissue-specific replication of Leishmania chagasi. J Immunol 156: 2231–2239.
43. Maroof A, Beattie L, Zubairi S, Svensson M, Stager S, et al. (2008) Posttranscriptional regulation of Il10 gene expression allows natural killer cells to express immunoregulatory function. Immunity 29: 295–305.
44. Veiga-Fernandes H, Coles MC, Foster KE, Patel A, Williams A, et al. (2007) Tyrosine kinase receptor RET is a key regulator of Peyer's patch organogenesis. Nature 446: 547–551.
45. Quah BJ, Barlow VP, McPhun V, Matthaei KI, Hulett MD, et al. (2008) Bystander B cells rapidly acquire antigen receptors from activated B cells by membrane transfer. Proc Natl Acad Sci U S A 105: 4259–4264.
46. Goodnow CC, Crosbie J, Adelstein S, Lavoie TB, Smith-Gill SJ, et al. (1988) Altered immunoglobulin expression and functional silencing of self-reactive B lymphocytes in transgenic mice. Nature 334: 676–682.
47. Beattie L, Evans KJ, Kaye PM, Smith DF (2008) Transgenic Leishmania and the immune response to infection. Parasite Immunol 30: 255–266.
48. Stager S, Alexander J, Carter KC, Brombacher F, Kaye PM (2003) Both interleukin-4 (IL-4) and IL-4 receptor alpha signaling contribute to the development of hepatic granulomas with optimal antileishmanial activity. Infect Immun 71: 4804–4807.
